# Genetic responsiveness of African buffalo to environmental stressors: A role for epigenetics in balancing autosomal and sex chromosome interactions?

**DOI:** 10.1371/journal.pone.0191481

**Published:** 2018-02-07

**Authors:** Pim van Hooft, Eric R. Dougherty, Wayne M. Getz, Barend J. Greyling, Bas J. Zwaan, Armanda D. S. Bastos

**Affiliations:** 1 Resource Ecology Group, Wageningen University, Wageningen, The Netherlands; 2 Mammal Research Institute, Department of Zoology & Entomology, University of Pretoria, Hatfield, South Africa; 3 Department of Environmental Science Policy & Management, University of California, Berkeley, California, United States of America; 4 School of Mathematical Sciences, University of KwaZulu-Natal, Durban, South Africa; 5 Agricultural Research Council, Irene, South Africa; 6 Laboratory of Genetics, Wageningen University, Wageningen, The Netherlands; Gaziosmanpasa University, TURKEY

## Abstract

In the African buffalo (*Syncerus caffer*) population of the Kruger National Park (South Africa) a primary sex-ratio distorter and a primary sex-ratio suppressor have been shown to occur on the Y chromosome. A subsequent autosomal microsatellite study indicated that two types of deleterious alleles with a negative effect on male body condition, but a positive effect on relative fitness when averaged across sexes and generations, occur genome-wide and at high frequencies in the same population. One type negatively affects body condition of both sexes, while the other acts antagonistically: it negatively affects male but positively affects female body condition. Here we show that high frequencies of male-deleterious alleles are attributable to Y-chromosomal distorter-suppressor pair activity and that these alleles are suppressed in individuals born after three dry pre-birth years, likely through epigenetic modification. Epigenetic suppression was indicated by statistical interactions between pre-birth rainfall, a proxy for parental body condition, and the phenotypic effect of homozygosity/heterozygosity status of microsatellites linked to male-deleterious alleles, while a role for the Y-chromosomal distorter-suppressor pair was indicated by between-sex genetic differences among pre-dispersal calves. We argue that suppression of male-deleterious alleles results in negative frequency-dependent selection of the Y distorter and suppressor; a prerequisite for a stable polymorphism of the Y distorter-suppressor pair. The Y distorter seems to be responsible for positive selection of male-deleterious alleles during resource-rich periods and the Y suppressor for positive selection of these alleles during resource-poor periods. Male-deleterious alleles were also associated with susceptibility to bovine tuberculosis, indicating that Kruger buffalo are sensitive to stressors such as diseases and droughts. We anticipate that future genetic studies on African buffalo will provide important new insights into gene fitness and epigenetic modification in the context of sex-ratio distortion and infectious disease dynamics.

## Introduction

Primary (at conception) and secondary (at birth) sex-ratio distortion in mammals can be both adaptive and non-adaptive. Mothers may adaptively and cryptically (e.g., *in utero*) preferentially invest in either sons or daughters when differences exist in their prospective lifetime reproductive success [1,2]. The best-known hypothesis of adaptive maternal sex allocation in mammals is the Trivers-Willard hypothesis [2]. According to this hypothesis, females in good condition or of high dominance (both measures reflecting maternal ability to invest in offspring) should favour sons because this is the sex that generally yields the highest marginal fitness returns [3]. Empirical support for the Trivers-Willard hypothesis has been reported, amongst others, for various ungulate species and humans [3,4]. The underlying physiological mechanism is thought to be maternal hormones, influencing sperm function and egg-receptivity in relation to sex of spermatozoa (X- or Y-bearing) in case of primary sex-ratio distortion and causing stress-related sex-specific foetal loss in case of secondary sex-ratio distortion [4,5]. In theory, fathers may adaptively and cryptically adjust the primary sex ratio by producing X- and Y-spermatozoa in unequal ratios or with different sperm competitive abilities [6]. However, reported paternal influences to date may also be a result of assortative mating according to body condition or dominance status [5].

Sex-ratio distortion can also be non-adaptive, generally with a reduction in fertility as a fitness cost [7–9]. This type of sex-ratio distortion occurs during spermatogenesis inside males and results from the occurrence of a sex-ratio distorter gene on one of the sex chromosomes that distorts meiosis in its own favour (meiotic drive) or impairs the function or viability of the opposite-sex spermatozoa [9]. Sex-ratio distorters exert strong selection pressure for suppressors of the distortion, which generally occur on the opposite sex chromosome [10]. Because selection occurs at a level below that of the individual, sex-ratio distorters can spread through a population even if they decrease the fitness of their carrier [11]. Interactions between sex-ratio distorters and suppressors resemble a coevolutionary arms race, which may result in complex dynamics, involving multiple sex-ratio distorters and suppressors together with enhancers and other modifiers [11]. A stable polymorphism of sex-ratio distorters and suppressors only seems possible through some form of negative frequency-dependent selection involving reduced fertility or survival as a fitness cost (without a cost to fitness sex-ratio distorters would quickly sweep to fixation) [9,11–16].

It has been shown that in the African buffalo (*Syncerus caffer*) of Kruger National Park both a sex-ratio distorter and a sex-ratio suppressor occur on the Y chromosome (Y distorter in haplotype 112 and Y suppressor in haplotype 557; frequency 112 = 0.051, 95% CI: [0.029,0.089], frequency 557 = 0.245, 95% CI: [0.193,0.307]) [15,17]. Significant associations with pre-birth rainfall and season of birth (dry vs. wet), which are direct proxies for parental body condition through their effects on resource availability, indicated that Y distorter activity is associated with high body condition (HBC), while Y suppressor activity is associated with low body condition (LBC). The Y distorter-suppressor pair has been hypothesized to be responsible for the observed male-biased sex ratio among wet-season-conceived foetuses conceptions and the observed female-biased sex ratio among dry-season-conceived foetuses [15]. Although the mechanism underlying negative frequency-dependent selection could not be deciphered, it was hypothesized that it involves the body-condition dependency of the Y distorter-suppressor pair [15].

Sex-ratio distorters can also impose fitness costs via deleterious alleles physically linked to the distorter [11]. High frequencies of deleterious alleles with a negative effect on male body condition were shown in the Kruger buffalo using autosomal microsatellites [18], although the male-deleterious alleles appeared to occur genome-wide and therefore not to be physically linked to the Y distorter-suppressor pair. Significant statistical associations between male-deleterious alleles on the one hand and the Y suppressor and pre-birth rainfall (high allele frequencies among animals born after dry years) on the other suggest a causal relationship between the Y distorter-suppressor pair and high frequencies of male-deleterious alleles [18].

Two types of male-deleterious alleles, each occurring within a different group of genes, were observed: 1) deleterious alleles with a negative effect on the body condition of both sexes; and 2) sexually antagonistic alleles with a negative effect on male body condition, but a positive effect on female body condition. These gene effects were observed only in individuals located south of the Olifants River in Kruger (southern Kruger). It was hypothesized that these differential effects were associated with the high prevalence of bovine tuberculosis (BTB) in southern Kruger and its near absence in northern Kruger at the time of sampling, especially considering that BTB in the Kruger buffalo is associated with low body condition [19,20]. At the time of sampling, in 1998, the disease was mainly restricted to southern Kruger, with a prevalence of 16–38%, while prevalence in northern Kruger was only 1.5% with all infected individuals sampled from a single herd just north of the Olifants River [21].

To explain their high frequencies, the male-deleterious alleles were hypothesized to be under positive selection due to a net positive relative fitness effect when averaged across sexes and generations. The alleles deleterious to both sexes seemed to be co-dominant and to have a relatively strong effect in males, although these observations were not statistically significant. Co-dominance of the deleterious alleles most likely is a prerequisite for their high frequencies as a consequence of Haldane’s Sieve (the bias against the establishment of recessive mutations) [22].

Associations with pre-birth rainfall may indicate not only that the male-deleterious alleles have an effect on parental fitness by affecting parental body condition but possibly also the occurrence of epigenetic modification [23–26], whereby parental body condition affects the expression of male-deleterious alleles in the offspring. This occurs either pre-conception through epigenetic modifications in the gametes (epigenetic inheritance *via* the gametes) or post-conception through developmental interactions between mother and offspring. Particularly early embryonic development is a critical period in the formation of epigenetic marks in the genome [23].

Positive selection of male-deleterious alleles hypothetically occurs because the Y suppressor in LBC males, which on average carry many of these alleles, prevents sex-ratio distortion that would otherwise result in a fertility decrease [7,9,11,16,18]. Further, body condition of male buffalo is thought to play a major role in their mating success [27]. In other words, LBC males, due to their low body condition, are hypothesized to have a relatively low mating success but, in the absence of non-adaptive sex-ratio distortion, experience high relative fertility. For the opposite reasons, HBC males probably have a relatively high mating success but low relative fertility. Positive selection of male-deleterious alleles can occur when the negative effect of sex-ratio distortion on male fertility is larger than the negative effect of the male-deleterious alleles on male reproductive success to the extent that the net effect on relative fitness is positive when averaged across sexes and generations. The occurrence of primary sex-ratio distortion provides an explanation for the relatively strong deleterious allele effects in males, considering that it only affects male gametes [18].

In this study, we reanalysed the autosomal microsatellite data from Kruger buffalo as published in Van Hooft et al. 2014 [18] to answer the following four questions:

1. Are the male-deleterious alleles indeed co-dominant, considering that this seems to be a prerequisite for their high frequencies, particularly when the alleles are deleterious to both sexes (high frequencies of sexually antagonistic alleles may also be explained by their positive fitness effect in females)?
2. Does the selective regime in northern Kruger differ from that in southern Kruger; and, if so, can this be attributed to the high BTB prevalence in the latter?
3. Is the expression of male-deleterious alleles related to parental body condition, which would indicate some form of epigenetic modification?
4. Can conditional expression of male-deleterious alleles result in negative frequency-dependent selection of the Y distorter and suppressor?

Here we found significant statistical support to affirmatively answer these four questions, using a series of logistic regressions, pairwise comparisons of sex differences among pre-dispersal animals (i.e., 0–1 year old calves), and region-specific regressions between genetic measures and pre-birth rainfall per annual cohort.

## Results

### Logistic regressions

Logistic regression analyses resulted in 25 models with an Evidence Ratio ≤ 2.5 (Table 1). Of these, 11 models contained only main factors (not part of an interaction) and interaction factors with *P*-values ≤ 0.1. When considering only the highest ranking model for each regression among the latter models, a genetic-measure by pre-birth-rainfall interaction term was observed three out of six times: southern females and males with body condition as dependent variable (females: *P* = 0.013, model 14 in Table 1, S1 Table; males: *P* = 0.018, model 11 in Table 1, S2 Table) and southern females with BTB as dependent variable (*P* = 0.0013, model 20 in Table 1, S3 Table). Pre-birth rainfall as a main factor was observed twice: southern males with BTB as dependent variable and northern females with body condition as dependent variable (southern males: *P* = 0.0055, model 17 in Table 1, S4 Table; northern females: *P* = 0.058, model 7 in Table 1, S5 Table). Two additional models with a genetic-measure by pre-birth-rainfall interaction term were observed when lower ranking models were considered: the second highest ranking model for the southern males with BTB as dependent variable (*P* = 0.101, model 18 in Table 1, S6 Table) and the fifth highest ranking model for the northern males with body condition as dependent variable (*P* = 0.097, model 5 in Table 1, S7 Table). All five interactions were of the same sign: a larger effect size with increasing pre-birth rainfall. In other words, genetic effects on body condition and BTB infection risk were mainly observed among animals born after wet periods. Rainfall in each of the three years before the birth year on average showed significant or near-significant interaction with the genetic measures (3^rd^ pre-birth year: *P* = 0.0012, 2^nd^ pre-birth year: *P* = 0.0028, 1^st^ pre-birth year: *P* = 0.051; S8 Table). This was not observed for rainfall in the 4^th^ pre-birth year, the birth year or the year thereafter (*P* > 0.36, S8 Table).

**Table 1:** Best-ranked logistic regression models with Evidence Ratio ≤ 2.5.

Candidate models		AIC_c_	ER
*Body condition northern Kruger*			
Males (*N*_HBC_ = 27, *N*_LBC_ = 22, EPV = 2.0)			
1	A−*	59.3	
2	A−* | HetDE+	59.9	1.4
3	A−* | NDVI+	60.3	1.7
4	A−* | PBR+ | HetDE− | HetDE.PBR−	60.5	1.9
5	A−^#^ | PBR+ | HetDE− | HetDE.PBR−^#^ | NDVI+^#^	60.8	2.1
6	A−* | HetDE+ | NDVI+*	60.8	2.1
Females (*N*_HBC_ = 43, *N*_LBC_ = 46, EPV = 3.6)			
7	A−*** | NDVI+* | PBR+^#^	85.3	
8	A−** | NDVI+* | PBR+* | HomDE−	86.5	1.8
9	A−* | NDVI+* | P−	87.0	2.3
10	A−*** | NDVI+^#^	87.1	2.4
*Body condition southern Kruger*			
Males (*N*_HBC_ = 42, *N*_LBC_ = 92, EPV = 3.2)			
11	BTB−* | S−* | PBR− | HomDE− | HomDE.PBR−* | A−^#^	128.9	
12	BTB−* | S−* | PBR− | HomDE− | HomDE.PBR−* | A− | HetDE−	130.0	1.7
13	BTB−* | S−* | PBR− | HomDE− | HomDE.PBR −**	130.2	1.9
Females (*N*_HBC_ = 48, *N*_LBC_ = 138, EPV = 3.4)			
14	A−** | S−*** | PBR− | HetSAE+ | HetSAE.PBR+* | HomSAE+* NDVI+* | HomDE−^#^	173.5	
15	A−* | S−*** | PBR− | HetSAE+ | HetSAE.PBR+* | HomSAE+* NDVI+^#^	174.1	1.4
16	A−** | S−*** | PBR− | HetSAE+ | HetSAE.PBR+* | HomSAE+* NDVI+* | HomDE− | HomDE.PBR+	175.2	2.4
*BTB southern Kruger*			
Males (*N*_BTB-pos_ = 38, *N*_BTB-neg_ = 95, EPV = 2.9)			
17	BC−* | S+** | PBR−**	131.6	
18	BC−* | S+** | PBR−** | HomSAE− | HomSAE.PBR+^#a^	132.9	1.9
19	BC−* | S+** | PBR−** | HomSAE−	133.4	2.5
Females (*N*_BTB-pos_ = 48, *N*_BTB-neg_ = 138, EPV = 3.4)			
20	NDVI+* | PBR− | HomSAE+ | HomSAE.PBR−**	195.3	
21	NDVI+^#^ | PBR− | HomSAE+ | HomSAE.PBR−** | P+	195.3	1.0
22	NDVI+^#^ | PBR− | HomSAE+ | HomSAE.PBR−** | P+^#^ | A−	196.6	2.0
23	NDVI+* | PBR− | HomSAE+ | HomSAE.PBR−** | HetDE−	196.9	2.3
24	NDVI+^#^ | PBR− | HomSAE+ | HomSAE.PBR−** | HomDE+	196.9	2.3
25	NDVI+^#^ | PBR− | HomSAE+ | HomSAE.PBR−** | HomDE+ | P+	197.0	2.4

A: Age, BTB: bovine tuberculosis (categorical: BTB-negative = 0, BTB-positive = 1), BC: body condition (categorical: LBC, low body condition = 0; HBC, high body condition = 1), EPV: number of events per predictor variable, NDVI: Normalized Difference Vegetation Index, ER: Evidence Ratio, P: pregnancy (categorical: non-pregnant = 0, pregnant = 1), PBR: pre-birth rainfall, S: Sabie River (categorical: north of the Sabie River = 0, south of the Sabie River = 1), HomDE/HetDE/HomSAE/HetSAE: homozygosity/heterozygosity of deleterious-effect (DE) and sexually-antagonistic effect (SAE) associated microsatellite alleles, genetic-measure.PBR: interaction between genetic measure and pre-birth rainfall.

+: positive effect

−: negative effect

#a: *P* = 0.101

#: *P* ≤ 0.1

*: *P* ≤ 0.05

**: *P* ≤ 0.01

***: *P* ≤ 0.001.

Underlined models in first column: *P*-values of all factors ≤ 0.1, except for main factors that are part of a significant (*P* ≤ 0.05) or near-significant (*P* ≤ 0.1) interaction term.

Effects on body condition or BTB infection status were observed for all four genetic measures, with each of them showing an interaction with pre-birth rainfall at least once. HomDE and HetDE (homozygosity/heterozygosity of microsatellites associated with deleterious effects) had a negative effect on male body condition with increasing pre-birth rainfall in respectively southern and northern Kruger (*P* = 0.018, model 11 in Table 1, S2 Table; *P* = 0.097, model 5 in Table 1, S7 Table). Further, HomDE showed a negative effect on female body condition in both northern and southern Kruger (model 8 and 14 in Table 1, S1 and S9 Tables), resulting in a near-significant effect for Kruger as a whole (northern Kruger: *P* = 0.30, southern Kruger: *P* = 0.096, combined *P* = 0.055). HomSAE (homozygosity of microsatellites associated with sexually antagonistic effects) was associated with decreased levels of BTB infection with increasing pre-birth rainfall in females (*P* = 0.0013, model 20 in Table 1, S3 Table) but increased levels in males (*P* = 0.101, model 18 in Table 1, S6 Table). In southern Kruger, HetSAE (heterozygosity of microsatellites associated with sexually antagonistic effects) after relatively wet years before birth and HomSAE (no significant interaction with rainfall) were associated with high female body condition (HomSAE: *P* = 0.021, HetSAE: *P* = 0.013, model 14 in Table 1, S1 Table) but had no observable effect on male body condition (not included in any of the models with ER ≤ 2.5).

The expected net effect of HomDE, HomSAE and HetSAE (including interaction term) on female body condition, as estimated by multiplying these parameters with their regression coefficients, was negative for only 16% (31/187) of the southern females. These were mostly (87%, 27/31) individuals born after pre-birth periods with less than 484 mm annual rainfall. However, despite the expected net positive effect on body condition, the HBC fraction of BTB-negative females was relatively low in the south and almost significantly lower than that of BTB-negative males (northern Kruger: fraction HBC females = 0.497, 95% CI: [0.417,0.576], *N*_individuals_ = 147; fraction HBC males = 0.559, 95% CI: [0.458,0.656], *N*_individuals_ = 93; southern Kruger: fraction HBC females = 0.275, 95% CI: [0.215,0.345], *N*_individuals_ = 178; fraction HBC males = 0.361, 95% CI: [0.281,0.451], *N*_individuals_ = 119; sex difference, Fisher’s exact test: northern Kruger: *P* = 0.36, southern Kruger: *P* = 0.13, combined probability: *P* = 0.083).

Age had a negative effect (*P* ≤ 0.05, model 1–4, 6–10, 14–16 in Table 1; *P* ≤ 0.10, model 5 and model 11 in Table 1) and, as expected, NDVI (Normalized Difference Vegetation Index) a positive effect on body condition (*P* ≤ 0.05, model 6–9, 14 and 16 in Table 1; *P* ≤ 0.10, model 5, 10 and 15 in Table 1). A negative association between BTB infection and body condition was only observed among males (*P* ≤ 0.05, model 11–13 and 17–19 in Table 1). NDVI was positively associated with BTB in females (*P* ≤ 0.05, model 20 and 23 in Table 1; *P* ≤ 0.10, model 21–22 and 24–25 in Table 1), which may have been due to a relatively high BTB prevalence south of the Sabie River, which is the region with the highest average NDVI. No significant effects of pregnancy were observed (*P* = 0.090, model 22 in Table 1; *P* > 0.14 model 9, 21 and 25 in Table 1).

The model outcomes are unlikely to be attributed to false positives due to overfitting [28], considering that they were consistent and in agreement with earlier studies (S1 Text). All interaction terms were of the same sign (Sign test: *P* = 0.063), and age and NDVI consistently showed respectively negative and positive associations with body condition. According to the combined probability test, the interaction of the genetic parameters with pre-birth rainfall was highly significant even when taken into account the large number of possible interaction terms considered (four interaction terms in each of six modelling scenarios; *P* = 0.010, combined probability multiplied by number of possible combinations of five significant or near-significant observations out of 24; S1 Text).

Average Hedges’ *g* (BTB-positive vs. BTB-negative and LBC vs. HBC in each sex) of the individual male-deleterious load (MDL_male_ and MDL_female_) in southern Kruger was 0.52 (range: [0.42,0.69]) among animals born after wet 3-year pre-birth periods (> 550 mm/year). However, average Hedges’ *g* was close to zero in southern Kruger among animals born after dry 3-year pre-birth periods (mean: -0.07, range: [-0.36,0.27]) and among animals in northern Kruger irrespective of the amount of pre-birth rainfall (LBC vs. HBC in each sex; mean: 0.005, range: [-0.37,0.23]). This shows that the male-deleterious alleles were mainly active after wet years and that the selective regimes differed between northern and southern Kruger. Hedge’s *g* was particularly high when contrasting LBC BTB-positive with HBC BTB-negative southern animals born after wet 3-year periods (males: 0.96, 95% CI: [-0.03,1.95], *P* = 0.069; females: 0.85, 95% CI: [0.18,1.51], *P* = 0.0036; combined probability: *P* = 0.00082). Detailed results of Hedges’ *g* analyses are given in S10 Table.

### Pairwise comparisons of genetic measures between male and female calves

Among 0–1 year old calves HomDE was significantly higher and HetDE and HomSAE significantly lower in males than in females (HomDE: *P* = 0.049, HetDE: *P* = 0.031, HomSAE: *P* = 0.026, HetSAE: *P* = 0.97; Table 2). The combined total sex difference across the four genetic measures was significant in Kruger as a whole and in northern Kruger (whole Kruger: *P* = 0.012; northern Kruger: *P* = 0.024, southern Kruger: *P* = 0.13; Table 2).

**Table 2:** Sex differences among calves in four genetic measures.

Region	Mean males	Mean females	Mean pairwise difference	*P*-value
*Whole Kruger* *N*_herds_ = 26, *N*_individuals_ = 148				
HomDE	0.543	0.479	0.064	0.049
HetDE	0.721	0.836	-0.115	0.031
HomSAE	0.354	0.415	-0.060	0.026
HetSAE	0.737	0.738	-0.002	0.97
Combined			0.242	0.012
*Northern Kruger* *N*_herds_ = 8, *N*_individuals_ = 41				
HomDE	0.534	0.466	0.068	0.18
HetDE	0.623	0.859	-0.236	0.065
HomSAE	0.277	0.368	-0.091	0.084
HetSAE	0.732	0.673	0.059	0.36
Combined			0.454	0.024
*Southern Kruger* *N*_*herds*_ *= 18*, *N*_*individuals*_ *= 107*				
HomDE	0.548	0.485	0.063	0.13
HetDE	0.764	0.826	-0.062	0.25
HomSAE	0.389	0.436	-0.047	0.14
HetSAE	0.739	0.768	-0.029	0.56
Combined			0.200	0.13

HomDE/HetDE/HomSAE/HetSAE: homozygosity/heterozygosity of deleterious-effect (DE) and sexually-antagonistic effect (SAE) associated microsatellite alleles. Mean males and mean females: mean value of the herd means, *P*-value: obtained by randomizing observed values of each genetic measure among individuals within herds, combined: summation of the absolute differences of each genetic measure.

Despite the sex difference in three genetic measures, the fraction of HBC male and female BTB-negative calves was almost identical in both northern and southern Kruger (northern Kruger: fraction HBC females = 0.750, 95% CI: [0.606,0.854], *N*_individuals_ = 44; fraction HBC males = 0.745, 95% CI: [0.605,0.847], *N*_individuals_ = 47; southern Kruger: fraction HBC females = 0.387, 95% CI: [0.276,0.512], *N*_individuals_ = 62; fraction HBC males = 0.400, 95% CI: [0.281,0.532], *N*_individuals_ = 55).

### Region-specific regressions between genetic measures and pre-birth rainfall per annual cohort

**Fig 1:**
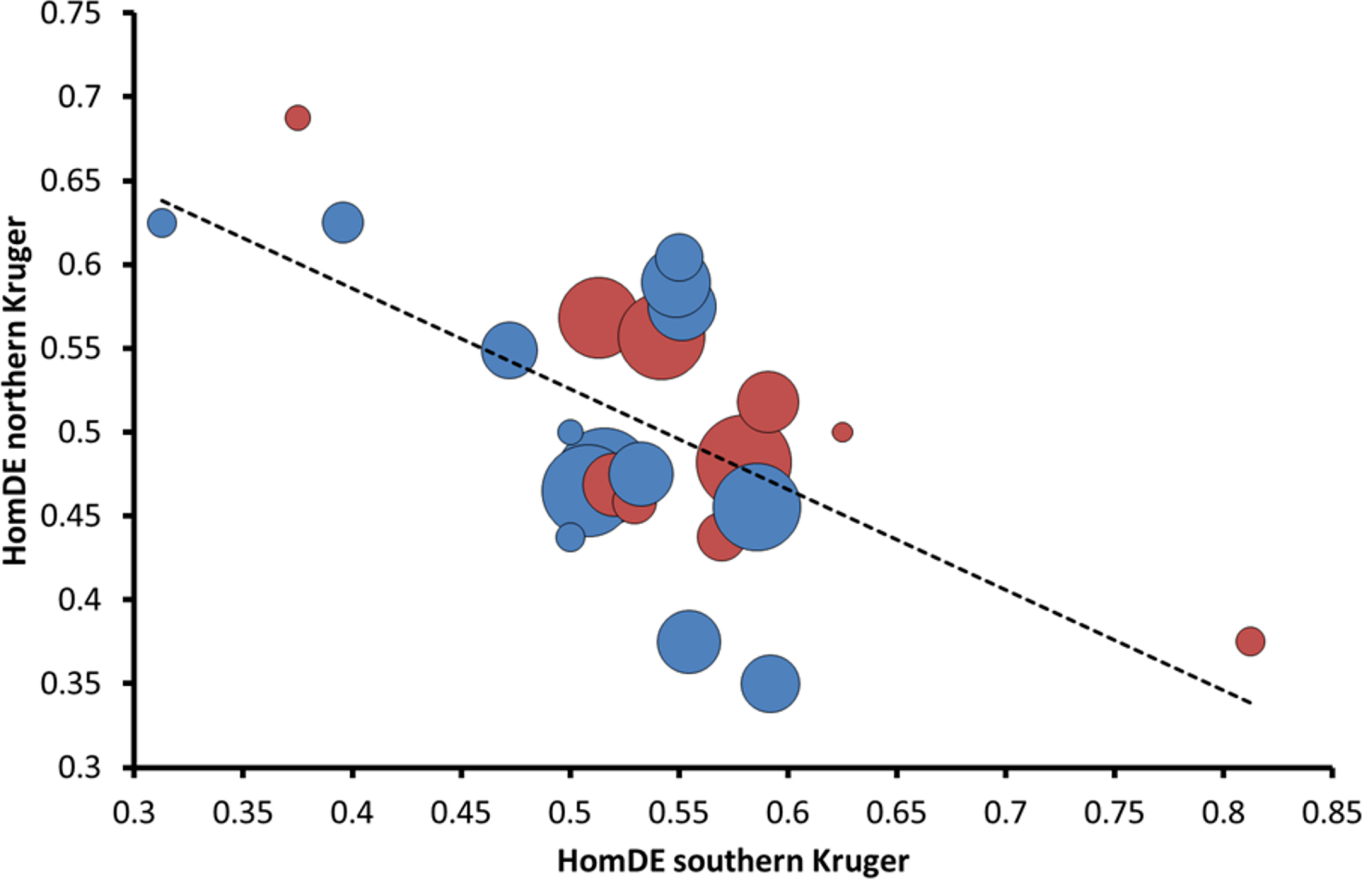
Negative correlation between northern and southern Kruger in HomDE per annual cohort. Circle size represents total number of sampled individuals (minimum = 2, maximum = 45). Blue circles: females, red circles: males. Females: Pearson *r* = -0.52, *P* = 0.054, *N*_annual-cohorts_ = 14; males: Pearson *r* = -0.73, *P* = 0.016, *N*_annual-cohorts_ = 10. HomDE: homozygosity of deleterious-effect (DE) associated microsatellite alleles.

There was a negative correlation for HomDE between northern and southern Kruger for cohorts of the same year, which was nearly significant for females and significant for males (females: Pearson *r* = -0.52, *P* = 0.054, *N*_annual-cohorts_ = 14; males: Pearson *r* = -0.73, *P* = 0.016, *N*_annual-cohorts_ = 10; sexes combined by averaging HomDE across sexes when possible, (males+females)/2: Pearson *r* = -0.69, *P* = 0.0066, *N*_annual-cohorts_ = 14; Fig 1). No significant correlations between northern and southern Kruger were observed for HomSAE, HetDE, or HetSAE (|Pearson *r*| < 0.54, *P* > 0.11). The negative correlation in HomDE suggests different selective regimes in northern Kruger, as was also indicated by the Hedges’ *g* analyses. The negative correlation suggests opposite responses to pre-birth rainfall in northern and southern Kruger. Indeed, although not significant per sex, regressions between HomDE and pre-birth rainfall were positive in the north, but negative in the south (northern Kruger: females: Pearson *r* = 0.28, *P* = 0.28, *N*_annual-cohorts_ = 17; males: Pearson *r* = 0.24, *P* = 0.45, *N*_annual-cohorts_ = 12; southern Kruger: females: Pearson *r* = -0.44, *P* = 0.10, *N*_annual-cohorts_ = 15; males: Pearson *r* = -0.23, *N*_annual-cohorts_ = 12, *P* = 0.47). Overall, there was a significant opposite regression between northern and southern Kruger (Fisher *r*-to-*z* transformation: females: *P* = 0.055, males: *P* = 0.23, combined probability: *P* = 0.027).

## Discussion

### Co-dominance of both deleterious and sexually antagonistic alleles

Co-dominance of the alleles deleterious to both sexes was hypothesized in Van Hooft et al. 2014 [18] to be a prerequisite for their high frequencies as a consequence of Haldane’s Sieve (question 1) [22]. The near-significant effect of HetDE on the body condition of northern males in the logistic regression analyses (model 5 in Table 1) and the significant difference in HetDE between male and female calves (Table 2) indicate that the deleterious alleles in the Kruger buffalo are indeed co-dominant. The significance of HetSAE in the logistic regression analyses indicate that the sexually antagonistic alleles are also co-dominant. Co-dominance of the sexually antagonistic alleles was only observed in the females (although its absence in males could be a type II error). This would be in agreement with studies that have shown that genes underlying intra-locus sexual conflict often exhibit sex-specific differences in dominance, with the higher dominance in the sex in which the genes are favoured (in female Kruger buffalo: higher body condition and lower BTB infection risk) [29–31].

### Direct influence of sex-ratio distorters

Three out of four genetic measures showed a significant difference between male and female calves, which we attribute to non-Mendelian transmission, considering that sex-specific dispersal or mortality is highly unlikely. The calves consisted of pre-weaning, pre-dispersal animals and sex-specific dispersal would indicate that large geographical regions with deviating values of HomDE, HetDE and HomSAE in one of the sexes remained unsampled. A relatively high HomDE but low HetDE among male calves argues against sex-specific mortality. The latter would imply that the deleterious alleles increase male mortality when heterozygous but increase female mortality when homozygous. Further, alleles causing sex-specific calve mortality, while simultaneously having high frequencies and being under positive selection [18], would imply a strongly increased fitness at reproductive age in the other sex or among the survivors of the same sex. Also the absence of any appreciable difference in fraction of HBC animals between male and female calves in both northern and southern Kruger argues against a strong allelic effect at young age. Thus, ruling out sex-specific mortality and dispersal, the observed genotypic differences between male and female calves could only be caused by non-Mendelian transmission of parental alleles.

**Fig 2:**
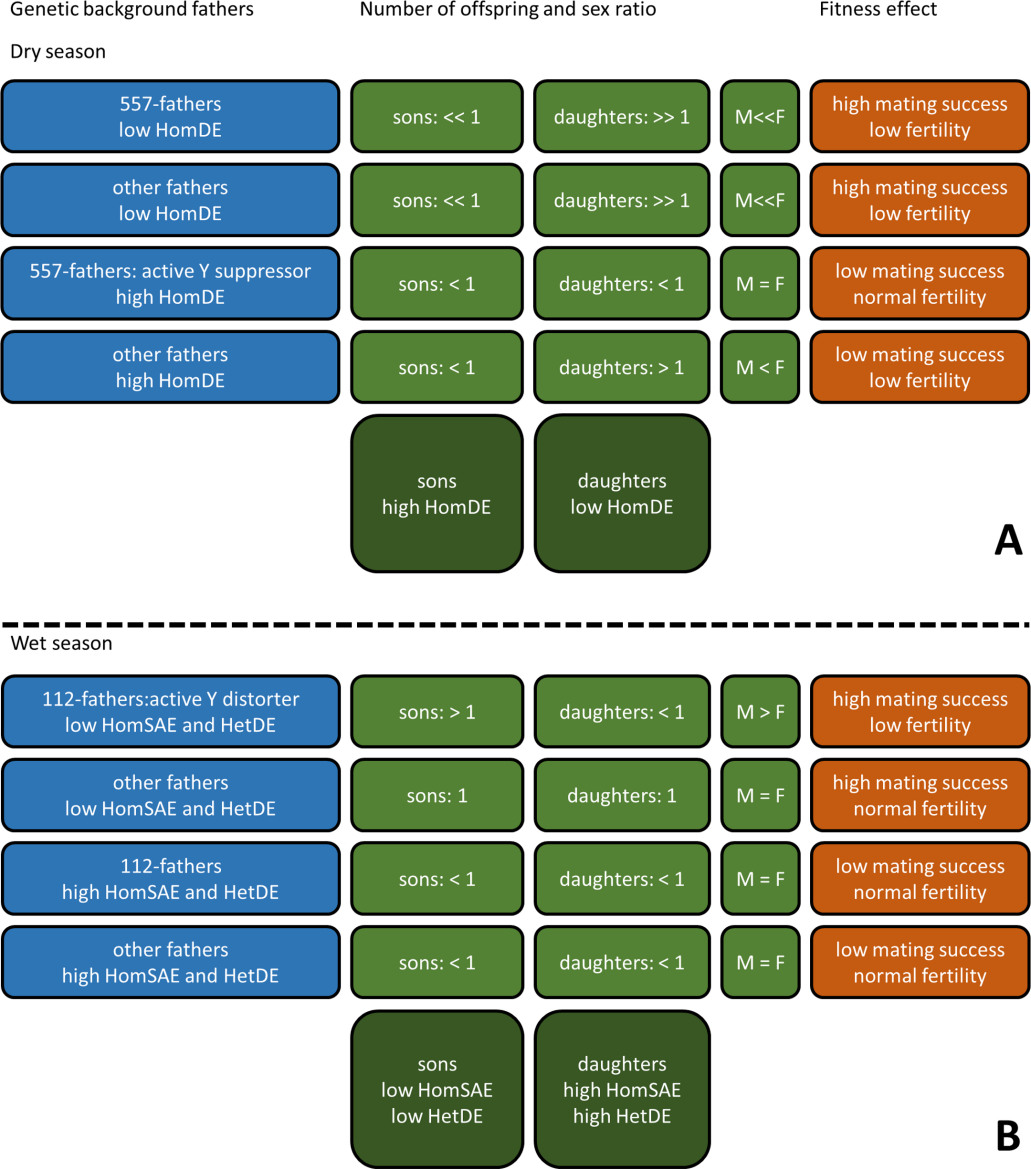
The hypothesized effect of seasonal Y distorter and suppressor activity on genetic differences between male and female calves. Offspring type = 1 (type is sons or daughters) is taken to represent replacement fitness (stationary population size). Offspring type < 1 and > 1 denote respectively a fitness cost and a fitness advantage due genetic effects on body condition (subsequently affecting mating success) and/or sex-ratio distortion (subsequently affecting fertility and relative number of offspring of one particular sex). Please note that for clarity this scheme assigns individuals to fixed low-high categories, although in reality the variables involved are continuous. We did not have data on mating success and fertility. It was hypothesized that high body condition has a positive effect on mating success [27] and sex-ratio distortion a negative effect on fertility [7,9]. Sex ratio in Kruger buffalo has been observed to be male-biased among foetuses conceived during wet seasons and female-biased among foetuses conceived during dry seasons [15]. This has been related to seasonal variation in Y distorter and suppressor activity, with the latter responding to a postulated X distorter [15]. (A) Dry season. Positive selection of male-deleterious alleles is possible when 557-fathers with high HomDE have the highest reproductive success (third row). Both the X distorter and the Y suppressor are assumed to be activated when body condition is low [15]. However, the relatively low HomDE among female calves suggests that the X distorter is easier activated by LBC than the Y suppressor (i.e. requiring less higher HomDE, first two rows). This results in a relatively strong sex-ratio distortion among offspring of fathers with low HomDE (first two rows). Among fathers with an active X distorter (rows one, two and four), those with low HomDE (first two rows) produce more daughters than those with high HomDE (fourth row) due to the high mating success of the former. This results in a relatively low HomDE among daughters (557-fathers with an active Y suppressor, and thus equal numbers of sons and daughters, do not contribute to the sex difference; third row). (B) Wet season. Positive selection of male-deleterious alleles is possible when 112-fathers with low HomSAE and HetDE, which results in HBC and thereby activates the Y distorter [15], have the lowest reproductive success, although they produce an above average number of sons due to sex-ratio distortion (first row). The latter results in a relatively low HomSAE and HetDE among all male calves (dry-season and wet-season conceived).

The most obvious cause of non-Mendelian transmission is the sex-ratio distorters that have been reported previously in the Kruger buffalo population [15,18], especially considering the earlier observation of a significant direct association between HomDE and a Y suppressor (Fig 6 in [18]). The Y suppressor in haplotype 557 was only observed among male calves born during a dry season, characterized by a female-biased primary sex ratio (deduced from observed sex ratios among dry-season-born calves and dry-season-conceived foetuses, with the gestation period being close to one year) [15]. The Y distorter in haplotype 112 was only observed among male calves born during a wet season, characterized by a male-biased primary sex ratio (deduced from observed sex ratios among wet-season-born calves and wet-season-conceived foetuses, with the gestation period being close to one year) [15]. Thus there was net selection for the Y suppressor during dry seasons and for the Y distorter during wet seasons [15]. The Y suppressor has been hypothesized to respond to an X distorter that causes the observed female-biased foetal sex ratio [15]. We think this seasonal contrast lies at the basis of the differences in HomDE, HomSAE and HetDE between male and female calves as hypothesized in Fig 2, which we now explain in detail.

Low HomDE among female calves can be attributed to individuals conceived during a dry season (Fig 2A), and low HetDE and HomSAE among male calves to individuals conceived during a wet season (Fig 2B). During dry seasons, LBC due to a high number of male-deleterious alleles is expected to activate the Y suppressor, thereby preventing a female-biased offspring sex ratio among suppressor-carrying males (third row in Fig 2A). Thus, fathers with many male-deleterious alleles (high HomDE) tend to produce about equal numbers of sons and daughters (slightly female-biased offspring because of high-HomDE fathers without a Y suppressor; last two rows in Fig 2A), while those with few male-deleterious alleles (low HomDE), generally give rise to daughters (first two rows in Fig 2A). The latter is due to the postulated X distorter whose activity also seems to be LBC-associated as female-biased foetal sex ratios have only been observed among dry-season-conceived foetuses [15]. However, the relatively low HomDE among female calves suggests that the X distorter is easier activated by LBC (at less higher HomDE) than the Y suppressor (first two rows in Fig 2A).

During wet seasons, HBC due to a low number of male-deleterious alleles is expected to activate the Y distorter (first row in Fig 2B). Consequently, fathers with few male-deleterious alleles (low HetDE and HomSAE) on average produce more sons than daughters (first two rows in Fig 2B). They produce more sons per individual than fathers with many male-deleterious alleles (last two rows in Fig 2B), although they are expected to have below average fertility because of the Y distorter.

The opposite effect of HomDE and HetDE may be attributed to a larger effect of HomDE than HetDE on male body condition because of co-dominance of the deleterious alleles. As a result, high HomDE tends to activate the Y suppressor during the dry season, much more so than high HetDE, while already moderate values of HomDE tend to inhibit the Y distorter during the wet season. Consequently, in male offspring HomDE is positively associated with a high paternal HomDE when conceived during the dry season and HetDE with a low paternal HetDE (activating the Y distorter) when conceived during the wet season.

According to the hypothesized mechanism depicted in Fig 2 positive selection can occur not only during dry resource-poor periods but also during wet resource-rich periods, with LBC-causing male-deleterious alleles preventing the activation of a sex-ratio distorter during both periods. During dry periods LBC indirectly prevents the activation of an X distorter via the Y suppressor (third row in Fig 2A) and during wet periods LBC directly prevents the activation of a Y distorter (third row in Fig 2B). In both circumstances, low male mating success, due to LBC and poor health (because of male-deleterious alleles), is offset by a high relative male fertility (not compromised by sex-ratio distortion). On the other hand, having few male-deleterious alleles results on average in a relatively high male mating success but low relative fertility (first two rows in Fig 2A and 2B); low fertility because of relatively few sons during dry periods (a consequence of the X distorter) and relatively few daughters during wet periods (a consequence of the Y distorter). Thus, it is the trade-off between mating success and relative fertility that increases the inclusive fitness of the male-deleterious alleles (analogous to the definition of inclusive fitness for individual organisms) [32].

### Different selective regimes in northern and southern Kruger

We expected the selective regime in northern Kruger to be different from that in southern Kruger due to the high BTB prevalence in the latter (question 2). According to the hypothesized mechanism in Fig 2, homozygous deleterious alleles (HomDE) experience the strongest positive selection during the dry seasons and homozygous sexually antagonistic alleles and heterozygous deleterious alleles (HomSAE and HetDE) during the wet seasons. Therefore, the negative correlation in HomDE between northern and southern year-cohorts with low HomDE in northern Kruger after dry years, the large sex difference in HomSAE and HetDE among northern calves and the small genetic effect on body condition in the north (Hedges’ *g*) suggest that in northern Kruger positive selection was relatively strong during wet seasons and in southern Kruger relatively strong during dry seasons, particularly after dry years.

The strong positive selection of homozygous deleterious alleles in southern Kruger was probably due to the high frequency of the Y suppressor in this part of the park, particularly considering the significant direct association between HomDE and the Y suppressor observed in an earlier study (frequency northern Kruger = 0.109, 95% CI: [0.054,0.209], frequency southern Kruger = 0.314, 95% CI: [0.242,0.396]; [18]). The high frequency of the Y suppressor in southern Kruger may have been a consequence of the locally high BTB prevalence. Logistic regression analyses indicated that BTB lowers male body condition, thereby activating the Y suppressor and increasing its selective advantage. This may have resulted in a positive feedback since the onset of the BTB epidemic in Kruger in the 1960s [33]. The reason, we argue, is that the male-deleterious alleles, by lowering body condition and increasing BTB infection risk among males, further increased the selective advantage of the Y suppressor, which subsequently increased the selective advantage of the male-deleterious alleles. The logistic regression analyses further suggested that the homozygous sexually antagonistic alleles (HomSAE) have a direct influence on BTB infection risk in both males (positive influence) and females (negative influence). An influence of genetic background on BTB infection status in southern Kruger was also indicated in another microsatellite study [34] and we suspect that male-deleterious alleles were involved here as well.

### Epigenetic modification and negative frequency-dependent selection of the Y distorter-suppressor pair

We tested whether the expression of male-deleterious alleles was related to parental body condition, which would be indicative of some form of epigenetic modification (question 3). For each of the four genetic measures, we observed an interaction with pre-birth rainfall in at least one logistic regression model. In all cases, a lower amount of pre-birth rainfall was associated with a smaller allelic effect size, which indicates that the male-deleterious alleles were suppressed in animals born after resource-poor dry periods when the average parental condition in the population is low. This points towards epigenetic modification, whereby body condition and health status of one or both parents influence expression of male-deleterious alleles in their offspring. The involvement of sex-ratio distorters that affect reproductive performance of only the fathers may suggest that this occurs through epigenetic modifications in the Y-spermatozoa (cellular epigenetic inheritance or epigenetic inheritance in the narrow sense). On the other hand, recent reviews indicated that epigenetic inheritance via the gametes may be rare with few studies convincingly excluding the possibility of intrauterine exposure, which would point towards epigenetic modification during early embryonic development [26,35,36]. Since the deduction of epigenetic modification is only based on statistical interactions between pre-birth rainfall and allelic effect size, we can give no further specifics about the underlying physiological mechanism.

We explored whether conditional expression of male-deleterious alleles, as indicated by the interactions with pre-birth rainfall, can in theory result in negative frequency-dependent selection of the Y distorter-suppressor pair (question 4). Negative frequency-dependent selection is a prerequisite to prevent fixation of either the distorter or the suppressor [9,12–14]. We developed a qualitative argument that posits a role for negative feedbacks caused by epigenetic gene suppression. According to this argument, during resource-poor periods 557-fathers with many active male-deleterious alleles have the highest reproductive success because they tend to have an active Y suppressor, thereby preventing reduced fertility that would otherwise occur. However, the male-deleterious alleles of their offspring are suppressed. This results in a negative feedback: few sons will have an active Y suppressor themselves thereby decreasing their reproductive success during subsequent resource-poor periods. During resource-rich periods, 112-fathers with suppressed male-deleterious alleles produce most of the male offspring because they tend to have an active Y distorter (relatively many sons) and an increased mating success (because of few male-deleterious alleles). However, the male-deleterious alleles of their offspring are active. Again, this results in a negative feedback: few sons will have an active Y distorter themselves, thereby decreasing the number of male offspring they produce during subsequent resource-rich periods.

### Possible ecological consequences

There seem to be constraints to the extent of deleterious effects on male body condition and health. Deleterious effects on male body condition and health likely become evident during stressful periods, such as those caused by drought and disease outbreaks, which is consonant with the fact that, in 2001, average body condition was observed to be lower in southern Kruger but only significantly so at the end of the dry season [19]. Further, and more importantly, the male-deleterious alleles appear to have had a net positive effect on female body condition and health (i.e., being free of BTB), which suggests positive selection of these alleles is only possible when negative effects on male fitness are offset by positive effects on female fitness. Most probably the amount of sexual antagonism in homologous phenotypic traits is constrained, thereby putting a limit to the effect size of the male-deleterious alleles [37]. This limit has likely not yet been reached, considering that there is still positive selection of both deleterious and sexually antagonistic alleles.

The relatively low body condition of female buffalo both in southern Kruger and relative to males seems to be counterintuitive in light of the mechanism described above. The relatively high body condition of male buffalo can most likely be attributed to sexual segregation. Male buffalo alternate their time between breeding herds and small bachelor groups. Males in bachelor groups tend to have relatively high body condition because of higher quality food, less distance travelled per day, and less pressure to defend or compete for females [27].

The relatively low body condition of female buffalo in southern Kruger is more difficult to explain, especially considering the relatively high NDVI values in this part of the park, indicating high resource availability. A possible explanation is maternal effects during weaning due to low body condition and poor health of BTB-positive mothers, which is supported by an earlier observed reduction in calving success of BTB-positive mothers [38]. Alternatively, it may be that the performance of many BTB-negative females in BTB-infected herds was negatively affected, perhaps through less efficient foraging. The latter was supported by a significant correlation between BTB prevalence per herd and fraction HBC among BTB-negative females (Pearson *r* = -0.48, *N*_individuals_ = 21, *P* = 0.028; S2 Fig). Low body condition might also be the result of population-density-dependent effects on body condition or resource competition with other herbivores considering the relatively high animal densities in southern Kruger [39]. Further, other environmental factors may have influenced female body condition, particularly diseases. Relatively high prevalence in southern Kruger, next to BTB, has been observed for brucellosis (bacterial disease) and Rift Valley Fever (a vector-borne viral disease transmitted by mosquitoes) [40,41]. The former is associated with decreased body condition and increased mortality in buffalo and the latter causes death of new-born animals and abortion in ruminants. Just as with BTB, male-deleterious alleles may have contributed to the high prevalence of these diseases.

Although the relatively high prevalence of various diseases in southern Kruger may be coincidental, we think it is important to study the potential role of male-deleterious alleles herein not only in Kruger but also in other buffalo populations from southern Africa (Mozambique, Zimbabwe, Botswana and South Africa), considering that the DE and SAE alleles observed in this study have relatively high frequencies throughout the region (SAE alleles: BM1824, CSSM19, DIK020, ILSTS026 and SPS115: Kruger = 0.568, mean other populations = 0.412, DE alleles: BM4028, ETH010, ETH225, INRA006, INRA28, TGLA227 and TGLA263: Kruger = 0.718, mean other populations = 0.634; based on raw data from 14 populations in [42]).

### Conclusions

We have shown that male-deleterious alleles are probably co-dominant and have a large effect on the body condition and BTB infection risk of both male and female buffalo in Kruger. The high frequencies of male-deleterious alleles were likely a result of positive selection driven by a Y distorter-suppressor pair. The male-deleterious alleles were suppressed in animals conceived during resource-poor periods, probably through epigenetic modification. We reason that this resulted in negative frequency-dependent selection of the Y distorter-suppressor pair. A relatively high disease prevalence in southern Kruger, particularly of BTB but possibly of other diseases as well, probably resulted in different selective regimes in northern and southern Kruger. Male-deleterious alleles are expected to make the buffalo population more sensitive to stresses such as disease outbreaks and droughts. The results from this study indicate that the African buffalo is an ideal model species for studies on gene fitness and epigenetic modification in the context of sex-ratio distortion and infectious disease dynamics.

## Materials and methods

### Description of population and samples

Kruger National Park in South Africa is a 19,485 km^2^ wildlife reserve. Between 1984 and 1993 Kruger’s African buffalo population fluctuated around 25,000 ± 5,000 (mean ± SD rounded to nearest thousand, with annual culls of 1,000 to 3,000), but from 1994 onwards rose to around 37,000 individuals in 2010 under a much-reduced culling regime [41,43]. The culls were performed across the entire park and were random with respect to age, sex and body condition. The average breeding herd size is approximately 250 individuals [18].

BTB prevalence in the Kruger buffalo population has steadily increased since it was first confirmed in 1990 [33]. BTB probably entered the population around 1960 at the southern river boundary through contact with domestic cattle [33]. From 1991–1992 BTB was still only observed in southern Kruger (south of the Olifants River), with a herd prevalence of 4–27%. In 1998, the disease was still mainly restricted to southern Kruger, but herd prevalence had increased to 16–38%. In contrast, overall prevalence in northern Kruger (north of the Olifants River) was 1.5% with all infected individuals sampled from a single herd just north of the Olifants River [21]. In 2005, the prevalence increased to 28–45% in southern Kruger and the disease reached the northern-most boundary of the park [38,44].

The blood samples used in this study were by-products from BTB prevalence studies conducted from September to November in 1998 [20,21]. Molecular analyses on these samples have been performed previously [15,17,18]. This study does not include any new samples or molecular analyses. In 1998, ten herds from each of three geographical zones were sampled (map with sampling locations in S1 Fig): north of the Olifants River (northern Kuger); between Olifants and Sabie River; south of the Sabie River (latter two zones: southern Kruger). By sampling 20–30 animals per herd from a total of 30 herds the precision of the BTB prevalence estimate at the herd and population level was maximized, while keeping mortalities to a minimum. These animals were culled without regard to sex or age using techniques approved by Kruger National Park authorities [45]. Culling was based on the use of succinyldicholine chloride, a muscle-paralysing agent administered via dart-syringe from a helicopter.

A total of 459 individuals were selected for this study; 138 from northern Kruger (north of the Olifants River) and 321 from southern Kruger (south of the Olifants River). Age was estimated in years. The BTB status of each individual was determined using necropsy and histopathology [21]. Individuals were grouped into two body condition classes: low body condition (LBC) and high body condition (HBC). This grouping was based on a standardized index score that ranged from 0 to 5 (5 being the best condition), based on fat deposits along the back and rump (LBC: 0–3, HBC: 4; only 12 individuals with index score of 0–2 and no individuals with index score of 5) [46]. These two classes represent body condition at the end of the dry and beginning of the wet season. This body condition score technique has been shown to be highly reliable in African buffalo [47].

Positive selection of male-deleterious alleles was previously detected in this population based on autosomal microsatellite profiles [18] generated using a high-throughput approach in which 17 microsatellite loci, randomly chosen with respect to genomic location in cattle, are combined in three core multiplex PCRs [48]. Wright’s *F*-statistics showed only minor genetic differentiation among herds (mean and 95% CI: *F*_ST_ = 0.012 ± 0.004) and subpopulations (*F*_ST_ northern vs. southern Kruger = 0.005 ± 0.002), and no strong deviation from Hardy-Weinberg equilibrium within herds (*F*_IS_ = 0.021 ± 0.024) [18]. Eight microsatellites contained a majority allele (frequency > 0.63) that was associated with LBC, thereby indicating linkage of each of these microsatellites to some gene expressing a deleterious allele [18]. At the other nine microsatellites low expected heterozygosity was associated with LBC in males but HBC in females, indicating linkage to a gene expressing a sexually antagonistic allele [18]. We pooled the three most frequent alleles at each of the latter nine microsatellites because it was previously argued that these alleles can be considered to be most likely linked to the postulated sexually antagonistic allele as their high frequency indicated positive selection [18]. The occurrence of a significant allele cline based on these pooled alleles, with increasing frequencies from northern to southern Kruger, confirmed that they were indeed under positive selection [18]. The validity of allele pooling is further supported in the current study by four independent results based on this pooling (near-significance of HomSAE in males in logistic regression analysis, significance of HomSAE and HetSAE in females in logistic regression analysis, significance of HomDAE difference between male and female calves; see Results section). Henceforth, we refer to the eight majority alleles as deleterious-effect-associated alleles (DE alleles) and the nine pooled-threesomes as sexually-antagonistic-effect associated alleles (SAE alleles).

Annual rainfall data, with years running from November to October, from 19 rainfall stations throughout Kruger (ten north of the Olifants River, four between Olifants River and Sabie River, five south of the Sabie River) were obtained from the South African Weather Service and from SANParks Scientific Services (map with rainfall stations in S1 Fig). The period November-October was chosen because rainfall and NDVI (Normalized Difference Vegetation Index) tend to increase sharply in November and reach their peak in March [26]. Between November and March 78% of the annual rainfall occurs [15]. Mean annual rainfall in the three years before the year of birth was used as an estimate of pre-birth rainfall, because correlations with Y-chromosomal haplotype frequencies and expected heterozygosity at autosomal microsatellites were previously shown to be strongest for a 3-year period [15,17,18].

### Statistical analyses

In assessing the possible co-dominance of male-deleterious alleles, we defined three genotype classes per microsatellite: homozygotes with two deleterious-effect (DE) or sexually-antagonistic-effect (SAE) alleles (11), heterozygotes with one DE or SAE allele (01) and homozygotes without a DE or SAE allele (00). For each individual buffalo, we calculated the average homozygosity of its eight DE microsatellites and called this fractional value HomDE. HomDE = 0 indicates that none of the eight sites are homozygous for a DE allele, whereas HomDE = 1 indicates that all of the eight sites are homozygous for a DE allele. Similarly, we calculated the average homozygosity across an individual’s nine SAE microsatellites and called the resulting fractional value HomSAE. Additionally, we computed a measure of the average heterozygosity per individual that was independent of homozygosity: fraction of heterozygous DE or SAE alleles (01) among the microsatellites that were not homozygous for a DE or SAE allele (00 and 01, but not 11): HetDE and HetSAE. In particular, heterozygosity was calculated as the number of male-deleterious alleles minus twice the number of homozygous loci divided by total number of loci minus the number of homozygous loci. Missing single-locus data (3.8% of the data) were replaced by single-locus estimates of mean number of homozygous alleles and mean number of male-deleterious alleles per region (northern and southern Kruger).

The average number of microsatellites used for estimating HetDE and HetSAE in each individual was relatively low at 3.8 and 5.4, respectively, but they were, at best, weakly correlated with HomDE and HomSAE for the population as a whole or when grouped by sex. Specifically, Pearson correlations among the four genetic measures HomDE, HomSAE, HetDE, and HetSAE were small, with a significant departure from 0 only observed between HomDE and HomSAE among males. They yielded the following values across all individuals and also for males and females separately: all individuals, |*r*| < 0.076, *P* > 0.10, *N*_individuals_ = 457–459; males, |*r*| < 0.161, *P* > 0.19, *N*_individuals_ = 183, except for HomDE vs. HomSAE with *r* = 0.161: *P* = 0.030; females, |*r*| < 0.112, *P* > 0.06, *N*_individuals_ = 274–276).

In determining whether there is (i) a link between male-deleterious alleles and parental body condition, indicative of some form of epigenetic modification, and (ii) a role of sex-ratio distorters and suppressors herein, three types of statistical analysis (detailed below) were performed that tested for a parental influence on the genetic make-up of the offspring:

1. Multiple logistic regressions using body condition score or BTB status as the single dependent variable and considering the four genetic measures and derived environmental factors as candidate independent variables. Genetic-measure by pre-birth-rainfall interactions were also included in these regressions because significant interactions would indicate an influence of pre-birth rainfall on allelic effect size, probably through its influence on parental body condition. Note that rainfall is the major determinant of resource availability, which has been shown to coincide with the body condition of adult female buffalo in Kruger [49].
2. Pairwise comparisons in the four constructed genetic measures between 0–1 year old pre-dispersal male and female calves. Significant differences would most likely be due to differences among fathers in the relative number of male and female offspring, due to the activity of sex-ratio distorters and suppressors, in relation to the number of DE or SAE alleles that the fathers carry. Sex-specific mortality seems unlikely, as that would imply high calf mortality, which would be very difficult to reconcile with high male-deleterious allele frequencies and positive selection (but see Discussion section).
3. Region-specific regressions between genetic measures and pre-birth rainfall per annual cohort. Significantly different regressions in northern and southern Kruger would indicate different genetic responses to pre-birth rainfall.

### Logistic regressions

Multiple logistic regression analyses were performed with body condition (LBC = 0, HBC = 1) or BTB status (BTB-negative = 0, BTB-positive = 1) as the binomial dependent variable. HomDE, HetDE, HomSAE, HetSAE, age, body condition (when BTB status was the dependent variable), BTB status (when body condition was the dependent variable), pregnancy status (0 = not pregnant, 1 = pregnant), pre-birth rainfall (average rainfall in the three years before the year of birth), and NDVI were included as candidate fixed independent variables. Herd affiliation was also incorporated as a random intercept in a mixed modelling approach. Resource availability, for which NDVI is a proxy, is one of the main environmental variables affecting body condition [50]. In Kruger buffalo, female body condition and monthly births have been shown to correlate with NDVI [49,51]. NDVI values associated with each herd were obtained from the Vegetation Health Product (VHP) derived from NOAA’s Polar Operational Environmental Satellites (POES). Each value is based on averages over a 20 km circular buffer zone around the sampling locality of each herd (based on home range estimates in [51,52], which in Kruger vary between 90 and 250 km^2^) over a 3-year period preceding September 1998 (where the grid interval size of the VHP layer is ~4km with readings taken at a weekly frequency). To test for a possible effect of pre-birth rainfall on allelic effect size, we included two-way interactions between pre-birth rainfall and each of the four genetic measures.

The dataset was divided by both sex and geographical region such that four subsets were analysed: northern males, northern females, southern males, and southern females. This was deemed a reasonable division based on our prior knowledge that significant genetic effects were only evident in the southern region [18]. This may be indicative of different selective regimes in the two regions. In the regressions for southern Kruger, the distinction between north and south of the Sabie River was added as a dummy variable because the fraction of HBC animals was considerably lower and BTB prevalence considerably higher south than north of the Sabie River (fraction HBC: south of the Sabie River: 0.120, 95% CI: [0.0784,0.180], *N*_individuals_ = 158; north of the Sabie River: 0.438, 95% CI: [0.364,0.515], *N*_individuals_ = 162; BTB prevalence: south of the Sabie River: 0.392, 95% CI: [0.320,0.470], *N*_individuals_ = 158; north of the Sabie River: 0.149, 95% CI: [0.102,0.212], *N*_individuals_ = 161). Consequently, separate estimates of pre-birth rainfall were used for the three geographical regions, viz. northern Kruger north of the Olifants River, southern Kruger between Olifants River and Sabie River, and southern Kruger south of the Sabie River, by averaging precipitation values of the rainfall stations in each region. If the Sabie River distinction is not included as dummy variable, herd-level factors, such as NDVI, may produce false positives in the logistic regression analyses due to the strong north-south contrast and the relatively low number of degrees of freedom for herd-level factors (number of herds minus 2).

We performed an exhaustive search over all possible theoretical variable sets, and selected the models that best fitted the data using the corrected Akaike Information Criterion (AIC_c_). Interaction terms were always included together with their constituent main factors. To aid in regression model convergence, all continuous variables were scaled by subtracting the mean of each variable from each observation and dividing the result by the standard deviation of that variable. We only considered models with a Evidence Ratio ≤ 2.5 (a measure of how much more likely the best model is than the model under consideration) [53]. Six classes of models were analysed: (1) northern males with body condition as dependent variable (*N*_individuals_ = 49); (2) northern females with body condition as dependent variable (*N*_individuals_ = 89); (3) southern males with body condition as dependent variable (*N*_individuals_ = 134); (4) southern females with body condition as dependent variable (*N*_individuals_ = 186); (5) southern males with BTB as dependent variable (*N*_individuals_ = 133); and (6) southern females with BTB as dependent variable (*N*_individuals_ = 186). Models for northern Kruger with BTB as dependent variable were not performed because only three out of 138 animals from this region were BTB-positive.

In the six modelling scenarios, the number of events per predictor variable (EPV) varied between 2.0 and 3.6 (Table 1), which is lower than the recommended minimum of 5.0 [28]. This may result in false positive outcomes and overfitting. However, a false positive result is unlikely when independent models based on different non-overlapping data sets give consistent outcomes. We tested for consistency by combining the probabilities from each predictor variable in the different modelling scenarios with the weighted *Z*-transform test (S1 Text) [54].

To estimate the effect size of the genetic-measure by pre-birth-rainfall interactions, we used Hedges’ *g* together with its 95% CI (difference between two means divided by the pooled SD, corrected for small sample size, equations 14 and 17 in [55]). We calculated Hedges’ *g* of the genetic measures (MDL, see below) between LBC and HBC animals, and between BTB-positive and BTB-negative animals for dry and wet pre-birth periods separately, defined as < or > 450 mm mean annual rainfall in northern Kruger and < or > 550 mm mean annual rainfall in southern Kruger; a distinction which creates data pairs of about equal size. As an estimate for male-deleterious load per individual we pooled the four genetic measures in males as

$${MDL}_{male}=HomDE+0.5\times HetDE+HomSAE+0.5\times HetSAE$$

and in females as

$${MDL}_{female}=HomDE+0.5\times HetDE-HomSAE-0.5\times HetSAE$$

HetDE and HetSAE were multiplied by 0.5 because of the co-dominance assumption, while HomSAE and HetSAE were subtracted in females to take into account sexual antagonism [18]. To correct for population genetic structure, means of MDL_male_ and MDL_female_ per herd per sex were equalized to zero by mean subtraction. MDL_male_ and MDL_female_ did not significantly deviate from normality (Shapiro-Wilk test: males: *P* = 0.37, *N* = 183, females: *P* = 0.16, *N* = 276). We conservatively chose the highest SD in each group comparison to correct for unequal variances. Significances of Hedges’ *g* were estimated with the unequal variance *t*-test [56].

### Pairwise comparisons of genetic measures between male and female calves

We estimated the significance of the average pairwise sex difference per herd for each of the four genetic measures separately and for the four genetic measures combined by summing their absolute values. Significance estimates were obtained by permuting genotypes within herds among individuals of both sexes. Significance was estimated as the fraction of random data sets showing the same or larger absolute difference than the original data set, using 100,000 randomizations. Probability estimates were obtained for the whole of Kruger (*N*_individuals_ = 148, *N*_herds_ = 26), and for northern (*N*_individuals_ = 41, *N*_herds_ = 8) and southern Kruger (*N*_individuals_ = 107, *N*_herds_ = 18) separately.

### Region-specific regressions between genetic measures and pre-birth rainfall per annual cohort

We tested across annual cohorts for correlations of the four genetic measures between northern and southern Kruger using Pearson’s correlation coefficient weighted by the square root of the total sample size. The correlations were conducted separately for each sex. Additionally, we performed linear regressions on annual cohorts between the genetic measures and pre-birth rainfall (estimated per region by taking the average precipitation values of the rainfall stations) weighted by the square root of the sample size. These regressions were conducted for each sex in each region. The Fisher *r*-to-*z* transformation was applied to estimate the significance of the difference between two regression coefficients.

Ninety-five percent confidence intervals of binomial proportions were estimated following Wilson [57]. Probabilities of differences between two proportions were estimated with Fisher’s exact test. Probabilities of parameters from different logistic regression analyses were combined with the weighted *Z*-transform test [54], weighted by the square root of the number of events (the smaller of the number of 0’s and 1’s). Pearson correlations, unequal variance *t*-tests and Fisher’s exact tests were performed using SPSS 22. Logistic regression analyses and exhaustive search over all possible theoretical variables were implemented using respectively the ‘lme4’ (version 1.1.12) and ‘glmulti’ package in R [58,59]. All other tests were conducted in Excel 2010 and SPSS 22. All reported *P*-values are two-sided.

## Supporting information

S1 Fig Map with locations of the rainfall stations and the sampled herds. (DOCX)

S2 Fig Regression between fraction HBC among BTB-negative females and BTB prevalence per herd. (DOCX)

S1 Table Logistic regression southern females with body condition status as dependent variable (highest ranking model). (DOCX)

S2 Table Logistic regression southern males with body condition status as dependent variable (highest ranking model). (DOCX)

S3 Table Logistic regression southern females with BTB status as dependent variable (highest ranking model). (DOCX)

S4 Table Logistic regression southern males with BTB status as dependent variable (highest ranking model). (DOCX)

S5 Table Logistic regression northern females with body condition status as dependent variable (highest ranking model). (DOCX)

S6 Table Logistic regression southern males with BTB status as dependent variable (Evidence Ratio = 1.9). (DOCX)

S7 Table Logistic regression northern males with body condition status as dependent variable (Evidence Ratio = 2.1). (DOCX)

S8 Table Significance of the genetic-measure by annual-rainfall interaction per single year. (DOCX)

S9 Table Logistic regression northern females with body condition status as dependent variable (Evidence Ratio = 1.8). (DOCX)

S10 Table Results Hedges’ *g* analyses (group differences with respect to MDL_male_ and MDL_female_). (DOCX)

S1 Text Consistency of the model outcomes. (DOCX)

## Abbreviations

BTB: bovine tuberculosis
LBC: low body condition
HBC: high body condition
HetDE: heterozygosity of deleterious-effect associated microsatellite alleles
HetSAE: heterozygosity of sexually-antagonistic-effect associated microsatellite alleles
HomDE: homozygosity of deleterious-effect associated microsatellite alleles
HomSAE: homozygosity of sexually-antagonistic-effect associated microsatellite alleles
NDVI: Normalized Difference Vegetation Index
