# Supplementary material for: Genetic responsiveness of African buffalo to environmental stressors: A role for epigenetics in balancing autosomal and sex chromosome interactions?
Source: PLoS One. 2018 Feb 7;13(2):e0191481. doi: 10.1371/journal.pone.0191481 (PMC5802885; doi:10.1371/journal.pone.0191481)
Supplement: S1 Fig — (DOCX) [file pone.0191481.s001.docx]

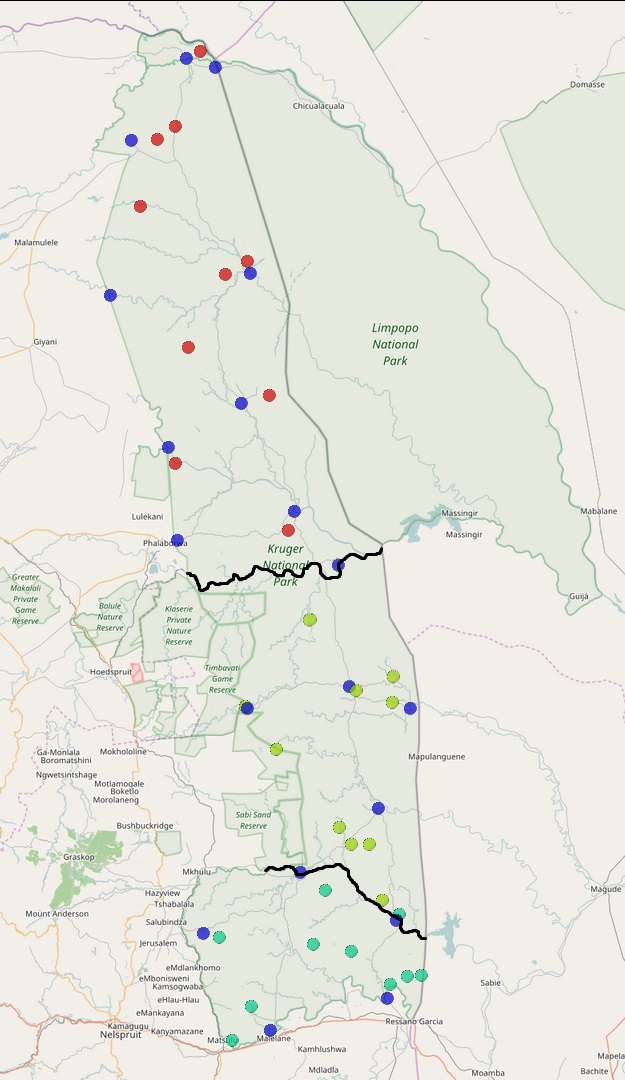


Figure S1: Map with locations of the rainfall stations and the sampled herds. Blue circles: rainfall stations, red circles: herds from northern Kruger, green circles: herds from southern Kruger north of the Sabie River, turquoise circles: herds from southern Kruger south of the Sabie River. The Olifants River in the centre and the Sabie River in the south are denoted in black.

© OpenStreetMap contributors. The data is available under the Open Database License, and the cartography is licensed as CC BY-SA.
