# Supplementary material for: Genetic responsiveness of African buffalo to environmental stressors: A role for epigenetics in balancing autosomal and sex chromosome interactions?
Source: PLoS One. 2018 Feb 7;13(2):e0191481. doi: 10.1371/journal.pone.0191481 (PMC5802885; doi:10.1371/journal.pone.0191481)
Supplement: S2 Fig — (DOCX) [file pone.0191481.s002.docx]

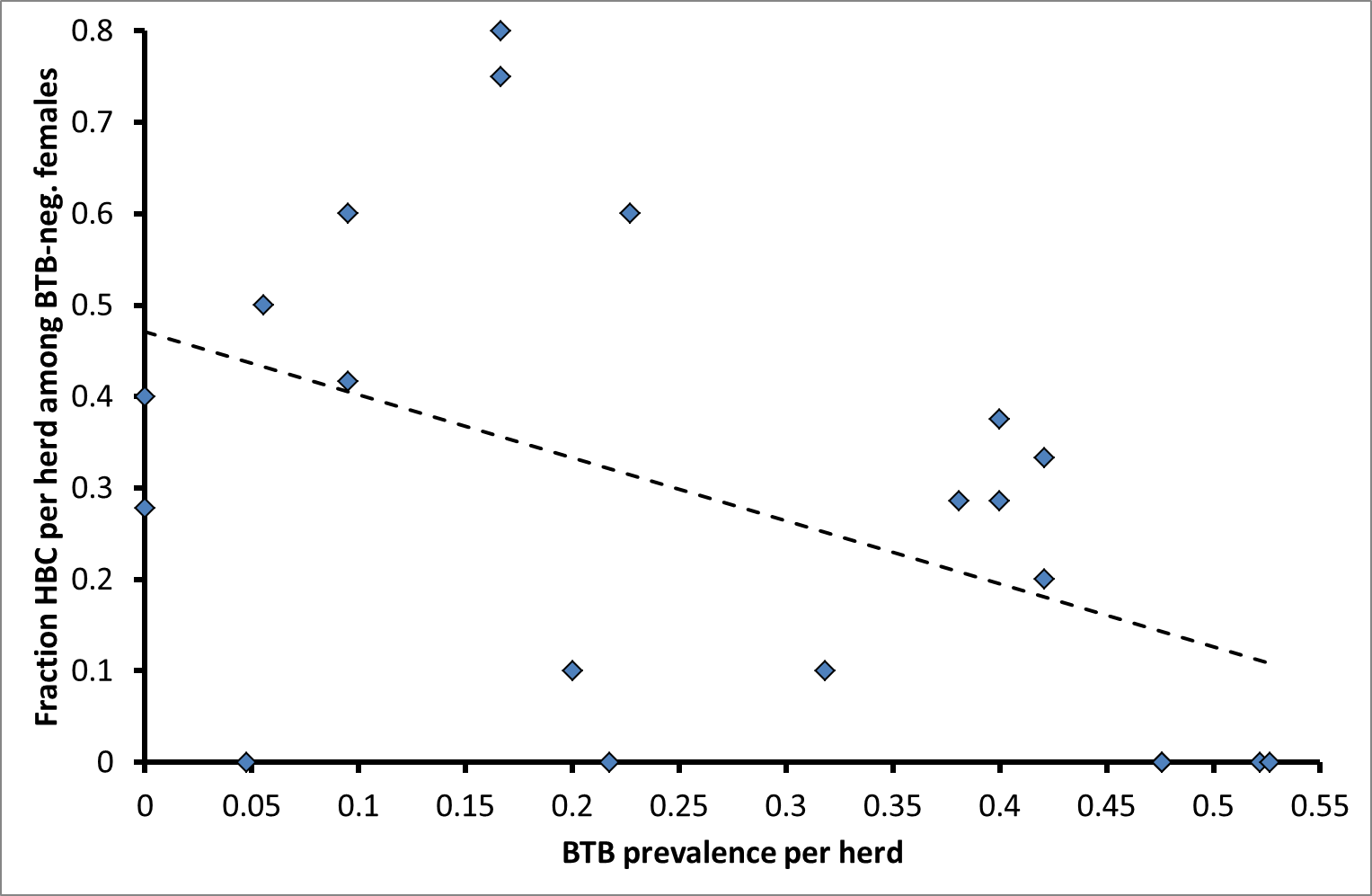


Figure S2: Regression between fraction HBC among BTB-negative females and BTB prevalence per herd

*N*_herds_ = 21 (20 from southern Kruger and 1 from northern Kruger), BTB prevalence per herd: *N*_individuals_ = 429, fraction HBC among BTB-negative females: *N*_individuals_ = 188. Adj. *R*^2^ = 0.19, *P* = 0.028. HBC: high body condition, BTB: Bovine tuberculosis
