# Supplementary material for: Genetic responsiveness of African buffalo to environmental stressors: A role for epigenetics in balancing autosomal and sex chromosome interactions?
Source: PLoS One. 2018 Feb 7;13(2):e0191481. doi: 10.1371/journal.pone.0191481 (PMC5802885; doi:10.1371/journal.pone.0191481)
Supplement: S1 Table — (DOCX) [file pone.0191481.s003.docx]

Table S1: Logistic regression southern females with body condition status as dependent variable (highest ranking model)

| Parameter | Unscaled estimate | Scaled estimate | SE | *P*-value |
| --- | --- | --- | --- | --- |
| Age | -0.168 | -0.677 | 0.252 | 0.0073 |
| NDVI | 29.870 | 1.145 | 0.559 | 0.041 |
| Sabie River (cat.) | -2.758 | -2.741 | 0.711 | 0.00012 |
| Pre-birth rainfall | -0.033 | -0.108 | 0.297 | 0.72 |
| HomDE | -2.304 | -0.400 | 0.240 | 0.096 |
| HomSAE | 3.744 | 0.590 | 0.257 | 0.021 |
| HetSAE | -20.713 | 0.256 | 0.249 | 0.30 |
| HetSAE*Pre-birth rainfall | 0.043 | 0.688 | 0.278 | 0.013 |
| Intercept | 4.409 | -1.032 | 0.408 | 0.011 |

Body condition: 0 = LBC (low body condition), 1 = HBC (high body condition), age: years, NDVI: 3-year period preceding September 1998 with a radius of 20 km, Sabie River, categorical variable: 0 = north of Sabie River, 1 = south of Sabie River, pre-birth rainfall: mean annual rainfall in the three years before the year of birth (mm/year), HomSAE/HomDE/HetSAE: homozygosity/heterozygosity of deleterious/sexually-antagonistic effect (DE/SAE) associated microsatellite alleles. Continuous variables were scaled by subtracting the mean of each variable from each observation and dividing the result by the standard deviation of that variable. SEs and *P*-values relate to the scaled estimates. *N*_LBC_=138, *N*_HBC_ = 48, *N*_herds_ = 20. Model 14 in Table 1.
