# Supplementary material for: Genetic responsiveness of African buffalo to environmental stressors: A role for epigenetics in balancing autosomal and sex chromosome interactions?
Source: PLoS One. 2018 Feb 7;13(2):e0191481. doi: 10.1371/journal.pone.0191481 (PMC5802885; doi:10.1371/journal.pone.0191481)
Supplement: S2 Table — (DOCX) [file pone.0191481.s004.docx]

Table S2: Logistic regression southern males with body condition status as dependent variable (highest ranking model)

| Parameter | Unscaled estimate | Scaled estimate | SE | *P*-value |
| --- | --- | --- | --- | --- |
| Age | -0.275 | -1.099 | 0.642 | 0.087 |
| BTB (cat.) | -1.634 | -1.634 | 0.796 | 0.040 |
| Sabie River (cat.) | -1.842 | -1.842 | 0.799 | 0.021 |
| Pre-birth rainfall | 0.038 | -0.732 | 0.590 | 0.21 |
| HomDE | 44.339 | -0.250 | 0.325 | 0.44 |
| HomDE*Pre-birth rainfall | -0.089 | -1.245 | 0.524 | 0.018 |
| Intercept | -17.679 | -0.045 | 0.540 | 0.93 |

Body condition: 0 = LBC (low body condition), 1 = HBC (high body condition), age: years, BTB, categorical variable: 0 = BTB-negative, 1 = BTB-positive, Sabie River, categorical variable: 0 = north of Sabie River, 1 = south of Sabie River, pre-birth rainfall: mean annual rainfall in the three years before the year of birth (mm/year), HomDE: homozygosity of deleterious-effect (DE) associated microsatellite alleles. Continuous variables were scaled by subtracting the mean of each variable from each observation and dividing the result by the standard deviation of that variable. SEs and *P*-values relate to the scaled estimates. *N*_LBC_=92, *N*_HBC_ = 42, *N*_herds_ = 20. Model 11 in Table 1.
