# Supplementary material for: Genetic responsiveness of African buffalo to environmental stressors: A role for epigenetics in balancing autosomal and sex chromosome interactions?
Source: PLoS One. 2018 Feb 7;13(2):e0191481. doi: 10.1371/journal.pone.0191481 (PMC5802885; doi:10.1371/journal.pone.0191481)
Supplement: S3 Table — (DOCX) [file pone.0191481.s005.docx]

Table S3: Logistic regression southern females with BTB status as dependent variable (highest ranking model)

| Parameter | Unscaled estimate | Scaled estimate | SE | *P*-value |
| --- | --- | --- | --- | --- |
| NDVI | 25.217 | 0.964 | 0.484 | 0.046 |
| Pre-birth rainfall | 0.029 | -0.164 | 0.252 | 0.52 |
| HomSAE | 40.577 | 0.128 | 0.223 | 0.56 |
| HomSAE*Pre-birth rainfall | -0.077 | -0.993 | 0.308 | 0.0013 |
| Intercept | -25.878 | -1.779 | 0.437 | <0.0001 |

BTB: 0 = BTB-negative, 1 = BTB-positive, NDVI: 3-year period preceding September 1998 with a radius of 20 km, pre-birth rainfall: mean annual rainfall in the three years before the year of birth (mm/year), HomSAE: homozygosity of sexually-antagonistic effect (SAE) associated microsatellite alleles. Continuous variables were scaled by subtracting the mean of each variable from each observation and dividing the result by the standard deviation of that variable. SEs and *P*-values relate to the scaled estimates. *N*_BTB-pos._=138, *N*_BTB-neg._ = 48, *N*_herds_ = 20. Model 20 in Table 1.
