# Supplementary material for: Genetic responsiveness of African buffalo to environmental stressors: A role for epigenetics in balancing autosomal and sex chromosome interactions?
Source: PLoS One. 2018 Feb 7;13(2):e0191481. doi: 10.1371/journal.pone.0191481 (PMC5802885; doi:10.1371/journal.pone.0191481)
Supplement: S4 Table — (DOCX) [file pone.0191481.s006.docx]

Table S4: Logistic regression southern males with BTB status as dependent variable (highest ranking model)

| Parameter | Unscaled estimate | Scaled estimate | SE | *P*-value |
| --- | --- | --- | --- | --- |
| Body condition (cat.) | -1.499 | -1.499 | 0.704 | 0.033 |
| Sabie River (cat.) | 1.753 | 1.754 | 0.586 | 0.0028 |
| Pre-birth rainfall | -0.012 | -0.976 | 0.351 | 0.0055 |
| Intercept | 4.627 | -1.505 | 0.532 | 0.0047 |

BTB: 0 = BTB-negative, 1 = BTB-positive, body condition, categorical variable: 0 = LBC (low body condition), 1 = HBC (high body condition), Sabie River, categorical variable: 0 = north of Sabie River, 1 = south of Sabie River, pre-birth rainfall: mean annual rainfall in the three years before the year of birth (mm/year). Continuous variables were scaled by subtracting the mean of each variable from each observation and dividing the result by the standard deviation of that variable. SEs and *P*-values relate to the scaled estimates. *N*_BTB-pos._=95, *N*_BTB-neg._ = 38, *N*_herds_ = 20. Model 17 in Table 1.
