# Supplementary material for: Genetic responsiveness of African buffalo to environmental stressors: A role for epigenetics in balancing autosomal and sex chromosome interactions?
Source: PLoS One. 2018 Feb 7;13(2):e0191481. doi: 10.1371/journal.pone.0191481 (PMC5802885; doi:10.1371/journal.pone.0191481)
Supplement: S5 Table — (DOCX) [file pone.0191481.s007.docx]

Table S5: Logistic regression northern females with body condition status as dependent variable (highest ranking model)

| Parameter | Unscaled estimate | Scaled estimate | SE | *P*-value |
| --- | --- | --- | --- | --- |
| Age | -0.463 | -1.848 | 0.546 | 0.00072 |
| NDVI | 176.223 | 7.022 | 3.416 | 0.040 |
| Pre-birth rainfall | 0.011 | 0.874 | 0.461 | 0.058 |
| Intercept | -56.700 | 10.363 | 4.503 | 0.021 |

Body condition: 0 = LBC (low body condition), 1 = HBC (high body condition), age: years, NDVI: 3-year period preceding September 1998 with a radius of 20 km, pre-birth rainfall: mean annual rainfall in the three years before the year of birth (mm/year). Continuous variables were scaled by subtracting the mean of each variable from each observation and dividing the result by the standard deviation of that variable. SEs and *P*-values relate to the scaled estimates. *N*_LBC_=46, *N*_HBC_ = 43, *N*_herds_ = 10. Model 7 in Table 1.
