# Supplementary material for: Genetic responsiveness of African buffalo to environmental stressors: A role for epigenetics in balancing autosomal and sex chromosome interactions?
Source: PLoS One. 2018 Feb 7;13(2):e0191481. doi: 10.1371/journal.pone.0191481 (PMC5802885; doi:10.1371/journal.pone.0191481)
Supplement: S6 Table — (DOCX) [file pone.0191481.s008.docx]

Table S6: Logistic regression southern males with BTB status as dependent variable (Evidence Ratio = 1.9)

| Parameter | Unscaled estimate | Scaled estimate | SE | *P*-value |
| --- | --- | --- | --- | --- |
| Body condition (cat.) | -1.466 | -1.466 | 0.720 | 0.042 |
| Sabie River (cat.) | 1.907 | 1.907 | 0.655 | 0.0036 |
| Pre-birth rainfall | -0.032 | -1.227 | 0.402 | 0.0023 |
| HomSAE | -23.856 | -0.309 | 0.270 | 0.25 |
| HomSAE*Pre-birth rainfall | 0.043 | 0.547 | 0.333 | 0.101 |
| Intercept | 15.617 | -1.551 | 0.573 | 0.0068 |

BTB: 0 = BTB-negative, 1 = BTB-positive, body condition, categorical variable: 0 = LBC (low body condition), 1 = HBC (high body condition), Sabie River, categorical variable: 0 = north of Sabie River, 1 = south of Sabie River, pre-birth rainfall: mean annual rainfall in the three years before the year of birth (mm/year), HomSAE: homozygosity of sexually-antagonistic effect (SAE) associated microsatellite alleles. Continuous variables were scaled by subtracting the mean of each variable from each observation and dividing the result by the standard deviation of that variable. SEs and *P*-values relate to the scaled estimates. *N*_BTB-pos._=95, *N*_BTB-neg._ = 38, *N*_herds_ = 20. Model 18 in Table 1.
