# Supplementary material for: Genetic responsiveness of African buffalo to environmental stressors: A role for epigenetics in balancing autosomal and sex chromosome interactions?
Source: PLoS One. 2018 Feb 7;13(2):e0191481. doi: 10.1371/journal.pone.0191481 (PMC5802885; doi:10.1371/journal.pone.0191481)
Supplement: S8 Table — (DOCX) [file pone.0191481.s010.docx]

Table S8: Significance of the genetic-measure by annual-rainfall interaction per single year

| Regression model  in Table 1 | *P*-value  4^rd^ pre-birth year | *P*-value  3^rd^ pre-birth year | *P*-value  2^nd^ pre-birth year | *P*-value  1^st^ pre-birth year | *P*-value  birth year | *P*-value  1^st^ year  after birth | *N* |
| --- | --- | --- | --- | --- | --- | --- | --- |
| Model 5 | 0.14^-^ | 0.11 | 0.59 | 0.87 | 0.98 | 0.40 | 22 |
| Model 11 | 0.42 | 0.23 | 0.27 | 0.13 | 0.74^-^ | 0.75^-^ | 42 |
| Model 14 | 0.70 | 0.061 | 0.16 | 0.30 | 0.60^-^ | 0.79 | 48 |
| Model 18 | 0.43^-^ | 0.048 | 0.42 | 0.70^-^ | 0.63 | 0.71^-^ | 38 |
| Model 20 | 0.017 | 0.44 | 0.011 | 0.084 | 0.46^-^ | 0.36^-^ | 48 |
| Combined *P*-value | 0.37 | 0.0012 | 0.0028 | 0.051 | 0.58 | 0.72 |  |

Each regression model is the same as the respective model in Table 1, except that rainfall was estimated for a single year instead of a 3-year pre-birth period. Continuous variables were scaled by subtracting the mean of each variable from each observation and dividing the result by the standard deviation of that variable. All *P*-values are 2-sided. The combined *P*-values were estimated with the weighted *Z*-transform test, weighted by the square root of the number of events (*N*: the smaller of the number of 0’s and 1’s in the logistic regression analysis). A negative sign at the end of a *P*-value indicates a smaller effect size with increasing rainfall, in which case an opposite *Z*-value was used in calculating the total *Z*-value in the Z-transform test. The Z-method assumes statistical independence of each observation (interaction term), which is justified because the Pearson correlations between the four genetic measures were weak at best (Pearson *r* ≤ 0.16, Materials and Methods section).
