# Supplementary material for: Genetic responsiveness of African buffalo to environmental stressors: A role for epigenetics in balancing autosomal and sex chromosome interactions?
Source: PLoS One. 2018 Feb 7;13(2):e0191481. doi: 10.1371/journal.pone.0191481 (PMC5802885; doi:10.1371/journal.pone.0191481)
Supplement: S9 Table — (DOCX) [file pone.0191481.s011.docx]

Table S9: Logistic regression northern females with body condition status as dependent variable (Evidence Ratio = 1.8)

| Parameter | Unscaled estimate | Scaled estimate | SE | *P*-value |
| --- | --- | --- | --- | --- |
| Age | -0.458 | -1.831 | 0.563 | 0.0012 |
| NDVI | 177.661 | 7.079 | 3.557 | 0.047 |
| Pre-birth rainfall | 0.011 | 0.939 | 0.476 | 0.048 |
| HomDE | -2.434 | -0.416 | 0.404 | 0.30 |
| Intercept | -56.323 | 10.403 | 4.686 | 0.026 |

Body condition: 0 = LBC (low body condition), 1 = HBC (high body condition), age: years, NDVI: 3-year period preceding September 1998 with a radius of 20 km, pre-birth rainfall: mean annual rainfall in the three years before the year of birth (mm/year), HomDE: homozygosity of deleterious-effect (DE) associated microsatellite alleles. Continuous variables were scaled by subtracting the mean of each variable from each observation and dividing the result by the standard deviation of that variable. SEs and *P*-values relate to the scaled estimates. *N*_LBC_=46, *N*_HBC_ = 43, *N*_herds_ = 10. Model 8 in Table 1.
