# Supplementary material for: Genetic responsiveness of African buffalo to environmental stressors: A role for epigenetics in balancing autosomal and sex chromosome interactions?
Source: PLoS One. 2018 Feb 7;13(2):e0191481. doi: 10.1371/journal.pone.0191481 (PMC5802885; doi:10.1371/journal.pone.0191481)
Supplement: S10 Table — (DOCX) [file pone.0191481.s012.docx]

Table S10: Results Hedges’ *g* analyses (group differences with respect to MDL_male_ and MDL_female_)

| Group^1^ | Pre-birth rainfall^2^ | Grouping variable^3^ | Hedges’ *g*^4^ | 95% CI low | 95% CI high | Sample size  LBC/BTB+ HBC/BTB- | *P*-value^5^ |
| --- | --- | --- | --- | --- | --- | --- | --- |
| southern males | wet | body condition | 0.690 | 0.111 | 1.269 | 34-20 | 0.0054 |
| southern males | wet | BTB | 0.424 | -0.445 | 1.293 | 48-6 | 0.30 |
| southern females | wet | body condition | 0.491 | 0.033 | 0.950 | 66-27 | 0.017 |
| southern females | wet | BTB | 0.463 | -0.018 | 0.944 | 70-23 | 0.047 |
| southern males | dry | body condition | -0.083 | -0.580 | 0.414 | 58-22 | 0.69 |
| southern males | dry | BTB | -0.105 | -0.560 | 0.350 | 47-32 | 0.63 |
| southern females | dry | body condition | 0.274 | -0.219 | 0.767 | 72-21 | 0.27 |
| southern females | dry | BTB | -0.362 | -0.829 | 0.104 | 68-25 | 0.12 |
| northern males | wet | body condition | 0.065 | -0.833 | 0.963 | 8-15 | 0.88 |
| northern females | wet | body condition | 0.225 | -0.432 | 0.883 | 16-23 | 0.48 |
| northern males | dry | body condition | -0.366 | -1.175 | 0.444 | 14-12 | 0.33 |
| northern females | dry | body condition | 0.095 | -0.483 | 0.673 | 30-20 | 0.71 |
| southern males | wet | body condition and BTB | 0.961 | -0.030 | 1.951 | 6-20 | 0.069 |
| southern females | wet | body condition and BTB | 0.846 | 0.178 | 1.514 | 18-22 | 0.0036 |
| southern males | dry | body condition and BTB | -0.152 | -0.743 | 0.440 | 29-19 | 0.55 |
| southern females | dry | body condition and BTB | -0.133 | -0.832 | 0.565 | 19-15 | 0.67 |

Dependent variable males: MDL_male_ (HomDE + 0.5xHetDE + HomSAE + 0.5xHetSAE), dependent variable females: MDL_female_ (HomDE + 0.5xHetDE - HomSAE - 0.5xHetSAE), 1: northern/southern: north/south of Olifants River, 2: northern Kruger: < or > 450 mm, southern Kruger: < or > 550 mm, 3: body condition: LBC vs. HBC, BTB: BTB-positive vs. BTB-negative, body condition and BTB: LBC BTB-positive vs. HBC BTB-negative, 4: positive value: highest mean among LBC and BTB-positive individuals, 5: unequal variance *t*-test.
