# Supplementary material for: Genetic responsiveness of African buffalo to environmental stressors: A role for epigenetics in balancing autosomal and sex chromosome interactions?
Source: PLoS One. 2018 Feb 7;13(2):e0191481. doi: 10.1371/journal.pone.0191481 (PMC5802885; doi:10.1371/journal.pone.0191481)
Supplement: S1 Text — (DOCX) [file pone.0191481.s013.docx]

**Consistency of the model outcomes**

We observed a significant or near-significant interaction between pre-birth rainfall and one of the four genetic measures in five out of six logistic modelling scenarios (dependent variable body condition: northern males, northern females, southern males, southern females; dependent variable bovine-tuberculosis (BTB) status: southern males, southern females). All of these interactions were of the same sign: a relatively weak association between genetic measure and body condition or BTB status after dry years. The combined two-sided probability according to the weighted *Z*-method [1] is 0.010 (*P* = 2.38*10^-7^ * 24!/(5!*19!): here the factorial expression is the number of possible combinations of five significant or near-significant observations (four interaction terms in each of six logistic model scenarios were considered), weighted by the square root of the number of events (the smaller of the number of 0’s and 1’s) per scenario). The *Z*-method assumes statistical independence of each observation (interaction term), which is justified because the Pearson correlations between the four genetic measures were weak at best (Pearson *r* ≤ 0.16, Materials and Methods section).

In the different modelling scenarios, the number of events per predictor variable (EPV) varied between 2.0 and 3.6, which is lower than the recommended minimum of 5.0 [2]. The likelihood of false positives in these various modelling scenarios is strongly reduced by consistency of the outcomes and their agreement with earlier studies. The models considered together had an EPV of 11.4 with respect to body condition (both sexes, northern and southern Kruger) and 6.1 with respect to BTB (both sexes, southern Kruger).

All interaction terms were of the same sign (Sign test: *P* = 0.063) and the one modelling scenario without an interaction term did include pre-birth rainfall in the highest ranking model (*P* = 0.058, model 7 in Table 1, Table S5). In all cases age showed a negative association with body condition (combined probability: *P* < 0.0001), in agreement with a recent study on 28 Kruger buffalo culled in July 2005 (Table 1 in [3]). As expected [4,5], NDVI (Normalized Difference Vegetation Index) showed a positive association with body condition (combined probability: *P* = 0.0034; *P* = 0.00088*4: *4 because one model did not include NDVI). Further, each of the three pre-birth years had a significant effect (Table S8), as has also been observed in earlier studies with respect to Y-chromosomal haplotype 557 [6] and expected heterozygosity at autosomal microsatellites (Figure S5 in [7]). HomDE (homozygosity of deleterious-effect associated microsatellite alleles) showed a negative association with body condition in both sexes in southern Kruger. HomSAE (homozygosity of sexually-antagonistic-effect associated microsatellite alleles) showed contrasting associations for the two sexes with BTB status: a positive one in males and a negative one in females. This supports the observation in an earlier study of sexual antagonism of the same microsatellites with respect to body condition (a negative association with body condition in males but a positive one in females) [7], a result strengthened by the fact that there was no significant association between body-condition status and BTB status in southern females (fraction high-body-condition (HBC) among BTB-positive females = 0.20, *N* = 66, fraction HBC among BTB-negative females = 0.28, *N* = 178, Fisher’s exact test: *P* = 0.25).

*References*

1. Whitlock MC (2005) Combining probability from independent tests: the weighted Z-method is superior to Fisher's approach. J Evol Biol 18: 1368-1373.

2. Vittinghoff E, McCulloch CE (2007) Relaxing the rule of ten events per variable in logistic and Cox regression. Am J Epidemiol 165: 710-718.

3. Taylor WA, Skinner JD, Boomker J (2013) Nematodes of the small intestine of African buffaloes, Syncerus caffer, in the Kruger National Park, South Africa. Onderstepoort Journal of Veterinary Research 80: 4.

4. Ryan SJ, Knechtel CU, Getz WM (2007) Ecological cues, gestation length, and birth timing in African buffalo (*Syncerus caffer*). Behav Ecol 18: 635-644.

5. Ryan SJ, Knechtel CU, Getz WM (2006) Range and habitat selection of African buffalo in South Africa. J Wildl Manage 70: 764-776.

6. van Hooft P, Greyling BJ, Prins HHT, Getz WM, Jolles AE, et al. (2007) Selection at the Y chromosome of the African buffalo driven by rainfall. PLoS ONE 2: 10.1371/journal.pone.0001086.

7. van Hooft P, Greyling BJ, Getz WM, van Helden PD, Zwaan BJ, et al. (2014) Positive Selection of Deleterious Alleles through Interaction with a Sex-Ratio Suppressor Gene in African Buffalo: A Plausible New Mechanism for a High Frequency Anomaly. PLoS ONE 9: e111778.
